# Strong Association Between HIV Incidence and Herpes Simplex Virus Type 2 in Zambia and South Africa: Prospective Data From the HPTN 071 (PopART) Trial

**DOI:** 10.1093/ofid/ofae721

**Published:** 2024-12-14

**Authors:** J Bradley, S Floyd, E Piwowar-Manning, O Laeyendecker, O R Baker, N Bell-Mandla, J Bwalya, A Moore, S H Eshleman, D Donnell, P Bock, S Fidler, H Ayles, R J Hayes

**Affiliations:** International Statistics and Epidemiology Group, London School of Hygiene and Tropical Medicine, London, UK; International Statistics and Epidemiology Group, London School of Hygiene and Tropical Medicine, London, UK; School of Medicine, Johns Hopkins University, Baltimore, Maryland, USA; National Institute of Allergy and Infectious Diseases, National Institutes of Health, Baltimore, Maryland, USA; School of Medicine, Johns Hopkins University, Baltimore, Maryland, USA; Desmond Tutu TB Centre, Department of Paediatrics and Child Health, Stellenbosch University, Cape Town, South Africa; Zambart, School of Medicine, University of Zambia, Lusaka, Zambia; FHI 360, Durham, North Carolina, USA; School of Medicine, Johns Hopkins University, Baltimore, Maryland, USA; Fred Hutchinson Cancer Center, Seattle, Washington, USA; Desmond Tutu TB Centre, Department of Paediatrics and Child Health, Stellenbosch University, Cape Town, South Africa; HIV Clinical Trials Unit, Department of Medicine, Imperial College London, London, UK; Zambart, School of Medicine, University of Zambia, Lusaka, Zambia; Clinical Research Department, London School of Hygiene and Tropical Medicine, London, UK; International Statistics and Epidemiology Group, London School of Hygiene and Tropical Medicine, London, UK

**Keywords:** cofactor, HIV, HSV2, prospective, Southern Africa

## Abstract

**Background:**

Herpes simplex virus type 2 (HSV2) is an important cofactor for HIV acquisition and transmission. Associations between the infections are reexamined in longitudinal data from an HIV prevention trial.

**Methods:**

The HPTN 071 (PopART) trial evaluated a combination prevention intervention in 21 urban communities in Zambia and South Africa. HIV incidence was measured in a cohort of approximately 2000 adults (age, 18–44 years) selected randomly from each community and followed up for 36 months. Incidence of HSV2 infection was estimated, and the effects of risk factors were examined. The association between HIV incidence and HSV2 infection was examined at individual and community levels.

**Results:**

An overall 10 539 participants were HSV2 negative at baseline and retested after 36 months. Estimated HSV2 incidence was 5.4 per 100 person-years (95% CI, 5.0–5.7) for women and 2.9 per 100 person-years (95% CI, 2.6–3.2) for men. When compared with those remaining HSV2 negative, HIV incidence was higher in those who were HSV2 positive at baseline (women: adjusted rate ratio [aRR], 3.24 [95% CI, 2.50–4.20]; men: aRR, 2.57 [95% CI, 1.60–4.11]) and even higher in those who seroconverted to HSV2 during follow-up (women: aRR, 5.94 [95% CI, 4.42–7.98]; men: aRR, 8.37 [95% CI, 5.18–13.52]). At the community level, strong associations were seen between HIV incidence and HSV2 prevalence (*R*^2^ = 0.48, *P* < .001) and incidence (*R*^2^ = 0.36, *P* = .004).

**Conclusions:**

There were strong associations between HIV incidence and HSV2 prevalence and incidence at individual and community levels. HSV2 control could contribute to HIV prevention.

Herpes simplex virus type 2 (HSV2) is the main cause of genital herpes and the most common viral sexually transmitted infection globally, with an estimated 491 million people aged 15 to 49 years affected in 2016 [1]. In addition to the burden of morbidity associated with genital herpes, there is strong evidence that HSV2 infection is a biological cofactor for HIV acquisition and transmission [2, 3].

A systematic review of longitudinal studies in 2017 [2] found an almost 3-fold increase in HIV incidence among people with prevalent HSV2 infection, with an even stronger 5-fold increase in those with incident HSV2 infection. The stronger association with incident HSV2 infection likely reflects the greater frequency and degree of symptomatology and genital pathology following initial infection with HSV2, although shared risk factors occurring during the same period may also contribute. The cofactor effect of HSV2 on HIV is important, not just because of its magnitude, but because of the high prevalence of HSV2 in countries with generalized HIV epidemics. HSV2 is a lifelong infection, and prevalence increases with age, plateauing at >70% in those aged ≥30 years in some populations [4, 5]. Consequently, a high proportion of incident HIV infections may be attributable to HSV2. A modeling study found that HSV2 infection was responsible for an estimated 37% of new HIV infections globally and 43% in the African region [6].

We previously reported data on HIV and HSV2 associations from the baseline survey of the HPTN 071 (PopART) trial, carried out in 21 urban communities in Zambia and South Africa between 2013 and 2015 [7]. In that cross-sectional analysis, we found a >6-fold higher prevalence of HIV infection in those with HSV2 infection in both sexes. We also carried out an ecological analysis of associations between the infections at community level, and this showed an extremely strong linear association. However, we acknowledged a key limitation of this analysis, which was the difficulty in determining the direction of causation given the cross-sectional design.

We subsequently collected data on HIV and HSV2 incidence from trial participants, and this offers the opportunity for longitudinal analyses that avoid some of the limitations of the previous report. Here we report the incidence of HSV2 infection by age and sex in our study population, factors associated with HSV2 acquisition, and the association between HIV incidence and HSV2 infection at the individual and community levels.

## METHODS

### Study Design

The HPTN 071 (PopART) trial took place between 2013 and 2018 (ClinicalTrials.gov NCT01900977, registered 16 July 2013), and full details of the study design have been presented [8]. Briefly, 21 urban and periurban communities in Zambia and South Africa (each defined as the catchment population of a health facility) were randomly allocated to 3 study arms. Arm A received the full PopART intervention: universal annual home-based HIV testing and counseling provided by lay community health workers, who also assisted with referral, linkage to care, and adherence support for clients who were HIV positive, in addition to the offer of immediate initiation of antiretroviral therapy (ART) irrespective of CD4+ T-cell count at the local clinic. Arm B received the full PopART intervention except that ART was provided according to current national treatment guidelines. Arm C was a control arm that continued to receive HIV testing and ART initiation in accordance with current national standard of care. In 2016, all arm B and C clinics transitioned to providing ART irrespective of CD4+ T-cell count, in accordance with changing national guidelines; arm C communities continued to receive existing standard of care for HIV testing. The trial results concerning the primary outcome of HIV incidence have been reported [9].

The impact of the PopART intervention on HIV infection incidence was measured in a population cohort comprising a random sample of adults from the general population. A random sample of households was selected, and with the agreement of the household head, all adults aged 18 to 44 years residing in each selected household were enumerated, with 1 selected at random for enrollment in the population cohort. Data for the analysis presented in this article came from the baseline survey of the population cohort, which was performed between November 2013 and March 2015, and from 3 follow-up surveys, which took place 12, 24, and 36 months after baseline.

Following informed consent, data on sociodemographic, behavioral, and other variables were collected from population cohort participants at each visit by an interviewer-administered questionnaire on a handheld electronic data capture device. At the end of the interview, a blood sample was collected by a trained research nurse and transported to the laboratory for processing, storage, and analysis. All blood samples were tested for HIV. Samples taken at baseline were tested for HSV2, as were samples taken at 36 months if the baseline sample was HSV2 seronegative. Samples taken at 12 and 24 months from individuals who seroconverted to HSV2 were tested for HSV2 to better estimate the time of seroconversion. The participant was informed that testing would be performed at a later date for research purposes and that results would not be returned, but for those wishing to know their HIV status, the nurse offered an on-the-spot rapid HIV test using a finger-prick blood sample.

### Laboratory Methods

HIV and HSV2 status was determined by testing blood samples collected from consenting survey participants. To determine HIV status, blood samples underwent in-country analysis by a single fourth-generation assay (Architect HIV Ag/Ab Combo Assay; Abbott Diagnostics). Further testing was performed at the HPTN Laboratory Center. Samples that had reactive results on in-country analysis were tested with a second fourth-generation assay (GS HIV Combo Assay; Bio-Rad Laboratories). Samples with discrepant/discordant test results were tested with additional assays to determine HIV status. For HSV2 status, blood samples underwent in-country analysis by the Kalon HSV2 immunoglobulin G enzyme-linked immunosorbent assay (Kalon Biological), where the in-country laboratories’ performance was validated by the HPTN Laboratory Center [10]. The manufacturer's calibrator was run in duplicate on all plate runs in the study, and optical density units from the enzyme-linked immunosorbent assay plates were converted to index values. The manufacturer's index value cutoffs (<0.9, negative; 0.9–1.1, indeterminate; >1.1, positive) were used to assign sample results. For the current study, samples with an indeterminate result were considered negative for HSV2.

### Statistical Analysis

Rates of HSV2 seroconversion were calculated by age, sex, and country. It was assumed that each HSV2 or HIV seroconversion took place at the midpoint between the last observed negative result and the first positive result. A Poisson regression model was used to explore risk factors for HSV2 seroconversion at the individual level, stratified by sex, with adjustment for age group (18–24, 25–29, 30–34, 35–39, 40–44 years) and study community. A hierarchical approach was used for model fitting [11], with the effects of sociodemographic variables adjusted for other sociodemographic variables and the effects of behavioral and biological variables adjusted for all other variables. Variables were measured at all surveys, and for this analysis we used the most recent value available. We performed an ecological analysis to explore the association between HSV2 incidence and risk factors at the community level and examined whether differences in HSV2 incidence across communities could be explained on the basis of measured behavioral or other variables.

The association between HSV2 status and HIV incidence at the individual level was then explored in a Poisson regression model. HSV2 status was categorized as negative (at baseline and endline), prevalent (positive at baseline), and incident (negative at baseline and positive at endline). The analysis adjusted for age group, sex, study community (and hence country and study arm), and all potential confounding factors for this association that were recorded. Population attributable fractions (PAFs) were calculated by standard methods, and the bootstrap with 1000 replicates was used to obtain confidence intervals [12]. For those who seroconverted to HSV2 and HIV and in whom the order of seroconversion could be determined, an analysis of which seroconversion occurred first was carried out with a McNemar test. Finally, in a further ecological analysis, we explored the association between community-level HIV incidence and community-level HSV2 incidence and baseline prevalence.

For the community-level ecological analyses, community-level rates and covariates were standardized by applying age- and sex-specific values from each community to a population with an equal proportion of males and females and the age structure of the cohort as a whole.

### Ethical Considerations

All participants in the population cohort gave written informed consent. The study and all the procedures presently described were approved by the ethics committees of the London School of Hygiene and Tropical Medicine, the University of Zambia, and Stellenbosch University.

## RESULTS

An overall 38 691 adults aged 18 to 44 years were enrolled in the cohort during the baseline survey (19 750 in Zambia and 18 941 in South Africa), of whom 37 272 (96%) were tested for HSV2 seropositivity. A total of 20 289 (54%) were seronegative at baseline; of those, 10 539 (52%) were retested at the 3-year survey, 1 of whom was excluded because the date of birth was not recorded. The 10 538 individuals included in the analysis provided 32 338 years of follow-up time: 5944 (56%) were from Zambia and 4594 (44%) were from South Africa; 3968 (38%) were male.

### HSV2 Incidence by Age, Sex, and Country

**Figure 1.**
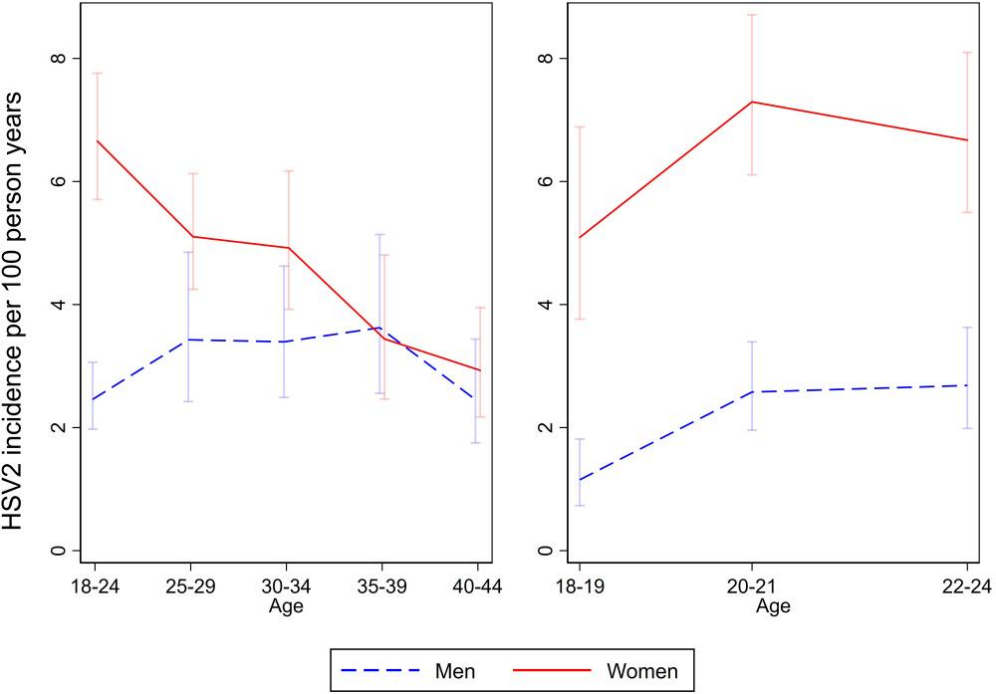
Herpes simplex virus type 2 (HSV2) incidence by sex in all age groups (left) and those aged <25 years (right). Vertical bars are 95% CIs.

There were 1425 HSV2 seroconversions in 32 338 person-years (PY) for an incidence of 4.4 seroconversions per 100 PY. The rate was considerably higher in women (5.5/100 PY, 1061/19 810; 95% CI, 5.0–5.7) than in men (2.9/100 PY, 364/12 528; 95% CI, 2.6–3.2). Incidence by age and sex is shown in Figure 1. For women the highest incidence was at age 20 to 21 years, and incidence consistently declined with older age. For men the association between incidence and age was much less pronounced, and incidence peaked at age 35 to 39 years. Incidence was higher in women than men in all age groups apart from those aged 35 to 39 years. Incidence was slightly higher in South Africa (4.9/100 PY, 698/14 106) than in Zambia (4.0/100 PY, 727/18 233).

### Risk Factors for HSV2 Incidence

Tables 1 and 2 show associations between sociodemographic and behavioral/biological variables and HSV2 incidence separately for men and women. After adjustment for age, community, and other factors, associations with several variables were observed in both sexes. HSV2 incidence increased with lower socioeconomic status in men and women and was also substantially higher in women who were divorced, separated, or widowed. Incidence increased steeply with reported lifetime number of sex partners and reported partners in the last year but was not associated with having a casual partner in the last year. Men who had medical circumcision had a lower incidence rate, but this was not observed in those reporting traditional circumcision. Baseline HIV infection was associated with higher HSV2 incidence in men and women. Marital status was not associated with HSV2 incidence in men, but married women had a lower incidence than those in other categories. Taking recreational drugs in the last year was associated with a higher incidence in women but not men.

**Table 1.** Risk Factors for Herpes Simplex Virus Type 2 Incidence Among Men in 21 Communities in Zambia and South Africa

Variable	Seroconversions/PY (Rate per 100 PY)	RR^a^ (95% CI)	aRR^b^ (95% CI)
**Overall**	364/12 528 (2.9)		
**Sociodemographic variables**			
Age, y		*P* = .116	*P* = .421
18–24	139/5655 (2.5)	1 [Reference]	1 [Reference]
25–29	95/2772 (3.4)	1.32 (1.01–1.71)	1.18 (.90–1.56)
30–34	57/1680 (3.4)	1.26 (.92–1.71)	1.12 (.80–1.56)
35–39	42/1159 (3.6)	1.43 (1.01–2.02)	1.14 (.78–1.67)
40–44	31/1263 (2.5)	1.00 (.67–1.48)	0.82 (.53–1.26)
Education		*P* = .756	*P* = .976
None/grades 1–2	4/92 (4.4)	1.49 (.55–4.06)	0.72 (.18–2.93)
Grades 3–6	15/497 (3.0)	0.99 (.58–1.71)	1.01 (.32–6.17)
Grades 7–10	136/4672 (2.9)	1 [Reference]	1 [Reference]
Grades 11–12	179/6164 (2.9)	0.89 (.71–1.11)	0.94 (.74–1.19)
College/university	23/907 (2.5)	0.89 (.56–1.39)	0.97 (.61–1.55)
Marital status		*P* = .072	*P* = .173
Married	122/4128 (3.0)	1 [Reference]	1 [Reference]
Never married	225/7942 (2.8)	0.79 (.61–1.03)	0.83 (.64–1.08)
Divorced/separated/widowed	17/458 (3.7)	1.33 (.79–2.22)	1.28 (.76–2.14)
Nights away in previous 3 mo		*P* = .881	*P* = .806
0	264/9145 (2.9)	1 [Reference]	1 [Reference]
1–6	57/1803 (3.2)	1.08 (.80–1.45)	1.10 (.82–1.49)
≥7	40/1506 (2.7)	1.03 (.72–1.46)	1.06 (.74–1.51)
Socioeconomic status^c^		*P* < .001	*P* < .001
1: Lowest	96/2085 (4.6)	1 [Reference]	1 [Reference]
2	103/2598 (4.0)	0.87 (.65–1.16)	0.88 (.65–1.18)
3	78/3599 (2.2)	0.50 (.36–.69)	0.51 (.37–.71)
4: Highest	78/4054 (1.9)	0.49 (.34–.70)	0.51 (.35–.74)
**Behavioral/biological variables**			
Baseline HIV status		*P* < .001	*P* < .001
Negative	321/12 147 (2.6)	1 [Reference]	1 [Reference]
Positive	43/382 (11.3)	3.89 (2.79–5.43)	4.14 (2.82–6.07)
Age at first sex, y		*P* = .105	*P* = .678
<16	105/2699 (3.9)	1 [Reference]	1 [Reference]
16–18	165/5489 (3.0)	0.84 (.65–1.07)	0.89 (.67–1.17)
>18	92/4282 (2.1)	0.73 (.54–.98)	0.88 (.62–1.27)
Lifetime sexual partners		*P* = .005	*P* = .021
0	10/1020 (1.0)	1 [Reference]	1 [Reference]
1	85/3342 (2.5)	2.20 (1.13–4.27)	2.22 (1.04–4.75)
2–4	113/4374 (2.6)	2.42 (1.26–4.64)	2.14 (1.01–4.57)
5–9	86/2437 (3.5)	2.81 (1.45–5.47)	2.78 (1.28–6.05)
≥10	62/1288 (4.8)	3.44 (1.73–6.83)	3.36 (1.51–7.50)
Sexual partners in the last year		*P* < .001	*P* < .001
0	9/897 (1.0)	1 [Reference]	1 [Reference]
1	208/7734 (2.7)	2.16 (1.10–4.24)	1.92 (.92–4.02)
≥2	94/2118 (4.4)	3.27 (1.63–6.57)	3.20 (1.47–6.94)
Casual sexual partner in the last year		*P* = .646	*P* = .249
No	286/9816 (2.9)	1 [Reference]	1 [Reference]
Yes	25/932 (2.7)	0.91 (.60–1.38)	0.77 (.49–1.21)
Condom use last sex		*P* = .678	*P* = .171
No	172/6655 (2.6)	1 [Reference]	1 [Reference]
Yes	167/4739 (3.5)	1.05 (.84–1.32)	0.83 (.64–1.08)
AUDIT score: alcohol use		*P* = .011	*P* = .057
0–7: Lower risk	292/10 487 (2.8)	1 [Reference]	1 [Reference]
8–15: Increasing risk	67/1693 (4.0)	1.43 (1.08–1.88)	1.32 (.97–1.78)
≥16: Higher risk/possible dependence	5/349 (1.4)	0.52 (.21–1.27)	0.53 (.21–1.31)
Ever taken recreational drugs		*P* = .686	*P* = .717
No	302/10 391 (2.9)	1 [Reference]	1 [Reference]
Yes	56/1963 (2.9)	1.06 (.79–1.43)	1.06 (.77–1.47)
Taken recreational drugs in last year		*P* = .430	*P* = .276
No	331/11 112 (3.0)	1 [Reference]	1 [Reference]
Yes	33/1417 (2.3)	0.86 (.59–1.25)	0.79 (.52–1.21)
Circumcision		*P* = .001	*P* = .004
Not circumcised	148/5907 (2.5)	1 [Reference]	1 [Reference]
Medically circumcised	58/3725 (1.6)	0.58 (.43–.79)	0.57 (.40–.81)
Traditionally circumcised	157/2892 (5.4)	1.13 (.80–1.59)	1.01 (.68–1.51)

Abbreviations: aRR, adjusted rate ratio; AUDIT, Alcohol Use Disorders Identification Test; PY, person-year; RR, rate ratio.

*P* values are derived from likelihood ratio tests.

^a^Adjusted for age group and community.

^b^Sociodemographic variables are adjusted for age group, community, and all other sociodemographic variables in this table. Behavioral/biological variables are adjusted for age group, community, and all other sociodemographic and behavioral/biological variables in this table, apart from the number of sex partners in the previous 12 months and lifetime sexual partners, which were not adjusted for each other because of collinearity.

^c^Quartiles of a wealth score based on household assets according to principal components analysis, separately for each country.

**Table 2.** Risk Factors for Herpes Simplex Virus Type 2 Infection Among Women in 21 Communities in Zambia and South Africa

Variable	Seroconversions/PY (Rate per 100 PY)	RR^a^ (95% CI)	aRR^b^ (95% CI)
**Overall**	1061/19 810 (5.4)		
**Sociodemographic variables**			
Age, y		*P* < .001	*P* < .001
18–24	552/8293 (6.7)	1 [Reference]	1 [Reference]
25–29	226/4490 (5.0)	0.69 (.59–.81)	0.81 (.69–.96)
30–34	155/3150 (4.9)	0.76 (.63–.90)	0.84 (.69–1.01)
35–39	68/1976 (3.4)	0.52 (.40–.67)	0.58 (.45–.76)
40–44	51/1829 (2.8)	0.48 (.36–.63)	0.51 (.38–.69)
Education		*P* = .335	*P* = .661
None/grades 1–2	17/343 (4.9)	1.06 (.65–1.73)	1.09 (.66–1.78)
Grades 3–6	57/1282 (4.4)	0.90 (.68–1.20)	0.89 (.67–1.18)
Grades 7–10	460/8380 (5.5)	1 [Reference]	1 [Reference]
Grades 11–12	457/8380 (5.5)	0.88 (.77–1.01)	0.92 (.80–1.06)
College/university	54/1153 (4.7)	0.81 (.61–1.08)	0.88 (.65–1.18)
Marital status		*P* < .001	*P* < .001
Married	428/10 652 (4.0)	1 [Reference]	1 [Reference]
Never married	518/7856 (6.6)	1.34 (1.16–1.56)	1.43 (1.22–1.66)
Divorced/separated/widowed	115/1302 (8.8)	2.30 (1.87–2.83)	2.26 (1.83–2.80)
Nights away in previous 3 mo		*P* = .897	*P* = .906
0	790/14 666 (5.4)	1 [Reference]	1 [Reference]
1–6	177/3343 (5.3)	0.97 (.82–1.15)	0.97 (.82–1.15)
≥7	90/1682 (5.4)	0.96 (.76–1.20)	0.96 (.76–1.21)
Socioeconomic status		*P* = .019	*P* = .028
1: Lowest	252/3803 (6.6)	1 [Reference]	1 [Reference]
2	247/4058 (6.1)	0.89 (.74–1.07)	0.90 (.75–1.08)
3	303/5501 (5.5)	0.88 (.73–1.07)	0.88 (.73–1.07)
4: Highest	243/6135 (4.0)	0.71 (.58–.89)	0.72 (.57–.90)
**Behavioral/biological variables**			
Baseline HIV status		*P* < .001	*P* < .001
Negative	935/18 859 (5.0)	1 [Reference]	1 [Reference]
Positive	126/951 (13.2)	2.49 (2.06–3.02)	1.99 (1.60–2.48)
Age at first sex, y		*P* < .001	*P* = .213
<16	155/2088 (7.4)	1 [Reference]	1 [Reference]
16–18	588/10 100 (5.8)	0.81 (.68–.97)	0.87 (.72–1.06)
>18	316/7582 (4.2)	0.64 (.53–.78)	0.82 (.66–1.02)
Lifetime sexual partners		*P* < .001	*P* < .001
0	28/1065 (2.6)	1 [Reference]	1 [Reference]
1	388/9930 (3.9)	1.73 (1.18–2.56)	1.81 (1.18–2.78)
2–4	536/7636 (7.0)	3.05 (2.07–4.48)	2.86 (1.86–4.38)
5–9	94/961 (9.8)	3.54 (2.30–5.47)	3.06 (1.89–4.94)
≥10	15/181 (8.3)	3.16 (1.67–5.98)	2.03 (1.01–4.09)
Sexual partners in the last year		*P* < .001	*P* < .001
0	26/1008 (2.6)	1 [Reference]	1 [Reference]
1	843/15 830 (5.3)	2.24 (1.51–3.33)	2.12 (1.39–3.23)
≥2	79/718 (11.0)	4.06 (2.58–6.40)	3.19 (1.96–5.21)
Casual sexual partner in the last year		*P* = .320	*P* = .729
No	916/16 876 (5.4)	1 [Reference]	1 [Reference]
Yes	32/679 (4.7)	1.21 (.83–1.75)	0.94 (.64–1.37)
Condom use last sex		*P* < .001	*P* = .409
No	618/13 409 (4.6)	1 [Reference]	1 [Reference]
Yes	371/4941 (7.5)	1.34 (1.16–1.53)	1.07 (.92–1.24)
AUDIT score: alcohol use		*P* = .005	*P* = .345
0–7: Lower risk	1008/19 138 (5.3)	1 [Reference]	1 [Reference]
8–15: Increasing risk	49/619 (7.9)	1.62 (1.21–2.17)	1.26 (.92–1.73)
≥16: Higher risk/possible dependence	4/53 (7.6)	1.33 (.50–3.56)	1.00 (.37–2.71)
Ever taken recreational drugs		*P* = .924	*P* = .228
No	1009/18 872 (5.3)	1 [Reference]	1 [Reference]
Yes	36/732 (4.9)	0.98 (.70–1.39)	0.78 (.53–1.17)
Taken recreational drugs in last year		*P* < .001	*P* < .001
No	1031/19 503 (5.3)	1 [Reference]	1 [Reference]
Yes	30/307 (9.8)	2.28 (1.57–3.31)	1.69 (1.10–2.61)

*P* values derived from likelihood ratio tests.

Abbreviations: aRR, adjusted rate ratio; AUDIT, Alcohol Use Disorders Identification Test; PY, person-year; RR, rate ratio.

^a^Adjusted for age group and community.

^b^Sociodemographic variables are adjusted for age group, community, and all other sociodemographic variables in this table. Behavioral/biological variables are adjusted for age group, community, and all other sociodemographic and behavioral/biological variables in this table, apart from the number of sex partners in the previous 12 months, and lifetime sexual partners which were not adjusted for each other because of collinearity.

### Ecological Analysis: Variation in HSV2 Incidence by Community

**Figure 2.**
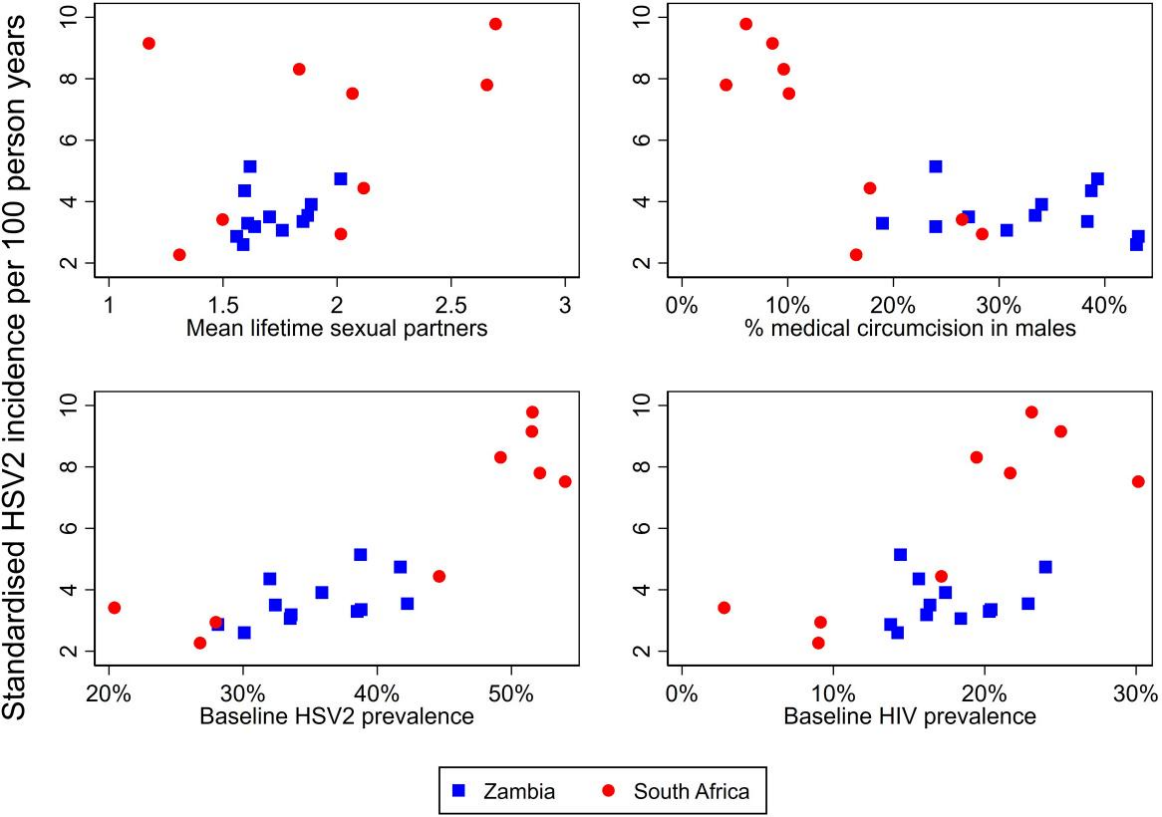
Association between community-level herpes simplex virus type 2 (HSV2) incidence and other community-level variables. Community-level rates and covariates are standardized by applying age- and sex-specific values from the community to a population with an equal proportion of males and females and the age structure of the cohort as a whole. Dots represent the 21 study communities.

The rate of HSV2 incidence varied from 2.3 to 9.8 seroconversions per 100 PY across the 21 communities. Figure 2 shows the association between community-level HSV2 incidence and other community-level variables. There was a strong relationship with medical male circumcision (*R*^2^ = 0.55, *P* < .001) and baseline HSV2 prevalence (*R*^2^ = 0.72, *P* < .001), but the association was weaker with baseline HIV prevalence (*R*^2^ = 0.37, *P* = .003) and mean number of lifetime sexual partners (*R*^2^ = 0.20, *P* = .003). A model with all 4 predictors had an *R*^2^ value of 0.81 (*P* < .001). Regression coefficients are given in Supplementary Table 1.

### Association Between HIV Incidence and HSV2 at the Individual Level

Table 3 shows the association between HIV incidence and HSV2 infection. In men and women, prevalent HSV2 infection was associated with a higher rate of HIV infection, but with an incident HSV2 infection, the rate of HIV infection was higher still. For these associations, there was only modest confounding by other variables. Overall PAFs for the proportions of HIV infections attributable to HSV2 were 48% in men and 62% in women.

**Table 3.** Association Between HSV2 Infection and HIV Incidence

HSV2 Status	HIV Seroconversions/PY (Rate per 100 PY)	RR^a^ (95% CI)	aRR^b^ (95% CI)	PAF^c^ (95% CI), %
**Men**		*P* < .001	*P* < .001	
Negative at baseline and 3-y follow-up	48/11 546 (0.4)	1 [Reference]	1 [Reference]	…
Prevalent: positive at baseline	49/5094 (1.0)	2.46 (1.59–3.79)	2.57 (1.60–4.11)	22 (10–33)
Incident: negative at baseline, positive at 3-y follow-up	40/1032 (3.9)	9.32 (6.04–14.38)	8.37 (5.18–13.52)	26 (16–35)
Total	…	…	…	48 (35–60)
**Women**		*P* < .001	*P* < .001	
Negative at baseline and 3-y follow-up	91/17 237 (0.5)	1 [Reference]	1 [Reference]	…
Prevalent: positive at baseline	416/24 860 (1.7)	3.35 (2.64–4.24)	3.24 (2.50–4.20)	45 (37–53)
Incident: negative at baseline, positive at 3-y follow-up	134/2918 (4.6)	7.53 (5.76–9.86)	5.94 (4.42–7.98)	17 (14–21)
Total	…	…	…	62 (54–71)

Abbreviations: aRR, adjusted rate ratio; AUDIT, Alcohol Use Disorders Identification Test; HSV2, herpes simplex virus type 2; PAF population attributable fraction; RR, rate ratio.

^a^Adjusted for age group and community.

^b^Adjusted for age group, community, education, marital status, number of nights away in previous 3 m, socioeconomic status, age at first sex, lifetime sexual partners, number of sexual partners in the last y, casual sexual partner in the last y, condom use last sex, AUDIT score, ever taken recreational drugs, taken recreational drugs in last y, and (for men) circumcision.

^c^Calculated from adjusted rate ratios.

### Timing of HIV and HSV2 Infection

Of the 174 participants who seroconverted to HIV and HSV2, there were 24 for whom the order of seroconversion could be determined (Supplementary Table 2). Of the 24, 17 seroconverted to HSV2 first (odds ratio, 2.43; 95% CI, 1.01–5.86; *P* = .047). All 24 participants reported already being sexually active at the time of enrollment in the population cohort.

### Ecological Analysis: Association Between Community-Level HSV2 and HIV

**Figure 3.**
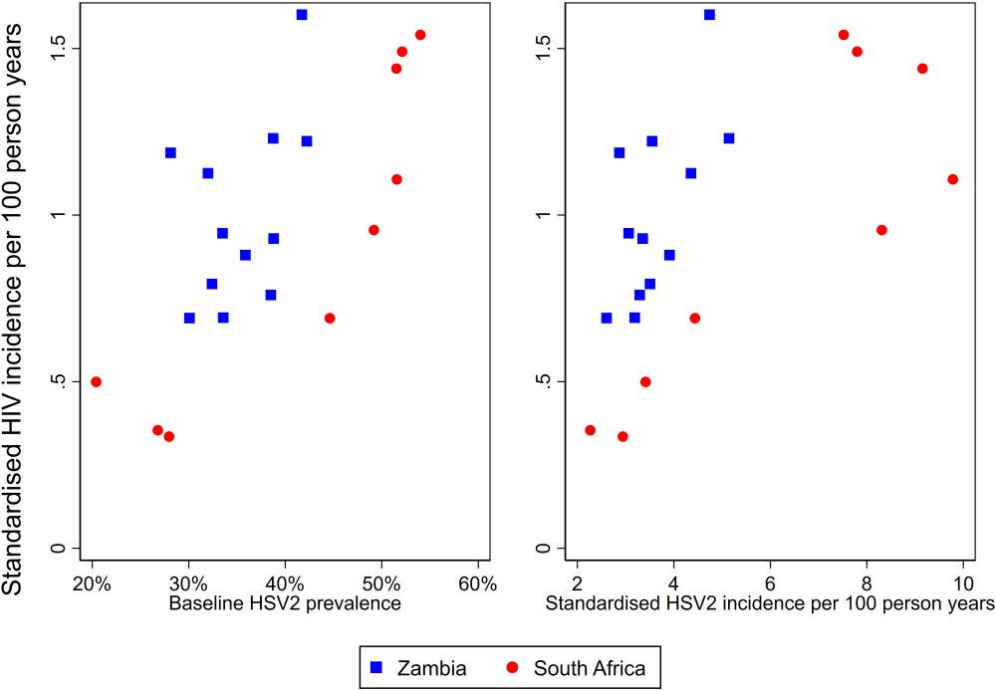
Association between community-level HIV incidence and community-level herpes simplex virus type 2 (HSV2) prevalence (left) and incidence (right). Dots represent the 21 study communities.

The community-level rate of HIV incidence varied from 0.34 to 1.60 per 100 PY. Figure 3 shows the association between community-level HIV incidence and community-level HSV2 prevalence and incidence. The association of HIV incidence with baseline HSV2 prevalence (*R*^2^ = 0.48, *P* < .001) was stronger than with HSV2 incidence (*R*^2^ = 0.36, *P* = .004).

## DISCUSSION

In this prospective study of a random sample of >10 000 adults who were initially HSV2 negative in 21 communities in Zambia and South Africa, we found a high incidence of HSV2 infection. The overall incidence of 4.4 per 100 PY in this cohort concealed large differences by age and sex. Incidence was higher in women (5.4/100 PY) than men (2.9/100 PY). These estimates are reasonably consistent with estimates from a systematic review that reported HSV2 incidence rates in the general population in Africa ranging from 3 to 21 per 100 PY in women (median, 8/100) and from 2 to 12 per 100 PY in men (median, 5/100) [13]. A more recent systematic review obtained similar results with incidence estimates (pooled means) of 7.2 per 100 PY in women and 4.1 per 100 PY in men [14]. Importantly, HSV2 is a lifelong infection, and so an incidence of around 4 per 100 PY implies that HSV2 prevalence increases with age to very high levels. This is consistent with many data from sub-Saharan Africa showing prevalence increasing with age to levels exceeding 50% to 60% in both sexes [4, 5, 15]. Higher observed HSV2 incidence in women than men, especially at younger ages, may reflect the age difference between sexual partners such that the male partners of women are often 5 to 10 years older [16–18].

In our study, HSV2 incidence peaked at around 8 per 100 PY in women aged 20 to 21 years, subsequently declining to <4 per 100 PY at older ages. There was less age-related variation in men. Previous studies have not shown consistent evidence of changes in HSV2 incidence with age [13, 14]. Moreover, any decline in incidence with age might reflect changes in sexual risk behavior in different age groups or a “frailty” effect whereby those at highest risk of HSV2 become infected at a younger age, leaving a residual population of individuals at lower risk [19]. In our previous study of HSV2 prevalence at the baseline survey of the HPTN 071 (PopART) trial, we used data on prevalence by age to obtain a rough estimate of HSV2 incidence [7]. Using this approach, we estimated HSV2 incidence to be 8.1 per 100 PY in women aged 18 to 24 years and 1.8 per 100 PY in men aged 18 to 24 years, and these estimates are very consistent with the rates of 6.7 and 2.5 per 100 PY measured directly in the present study.

We identified a number of risk factors for HSV2 incidence that broadly mirror factors associated with HSV2 prevalence in our previous study. Incidence was associated with lower socioeconomic status, number of sexual partners, and baseline HIV infection in both sexes and was higher in women who were divorced, widowed, or separated and who reported taking recreational drugs in the past year. These associations are likely to reflect differences between subgroups in their sexual behavior and/or the characteristics of sexual partners (including their HSV2 status), although it is plausible that HIV infection may also have a direct effect on susceptibility to HSV2 infection. HSV2 incidence was lower in men who were medically circumcised but not in those with traditional circumcision, and this is consistent with similar effects seen on HIV [18, 20, 21].

A major aim of the present study was to use our longitudinal data to examine the effect of HSV2 infection on HIV acquisition. Consistent with previous evidence from a meta-analysis [2], we found highly significant associations in both sexes, with a roughly 3-fold increase in incidence in those who were already HSV2 positive at baseline and a stronger 6- to 8-fold increase in those who seroconverted to HSV2 during the trial. These associations are likely to reflect, at least partly, a causal cofactor effect of HSV2 infection on susceptibility to HIV. This is reflected in the fact that, among those who seroconverted to both viruses, seroconversion to HSV2 was more likely to occur first. The stronger association with incident HSV2 infection may reflect the greater frequency and severity of symptoms of genital herpes that are observed shortly after infection. Associations were only slightly attenuated after adjusting for confounders, including numbers of sexual partners. The association of HIV incidence during follow-up with HSV2 infection at baseline also indicates the likely direction of causation. If we assume causality, our PAF estimates indicate that a very large proportion of HIV infections were attributable to HSV2, 48% in men and 62% in women. These estimates are consistent with PAF estimates for the African region based on mathematical modeling [6].

In our baseline study, we observed a very strong linear association between HSV2 prevalence and HIV prevalence at the community level [7]. A similar community-level association was seen for HIV incidence. Interestingly, HIV incidence at the community level was predicted more strongly by baseline HSV2 prevalence than by HSV2 incidence; however, these 2 variables were themselves strongly correlated, so it was difficult to separate out their effects. The stronger association with baseline prevalence may be explained by the higher PAF for prevalent infections observed in women.

Among the strengths of the present study were the large sample size and large numbers of seroconversions (for HSV2 and HIV); the availability of detailed prospective data on a range of sociodemographic, biological, and behavioral variables; and the collection of data from 21 communities with wide variations in HSV2 and HIV prevalence and incidence allowing for informative analysis of community-level correlations.

There were also several important limitations. First and most important, adequate control for confounding factors was clearly of key importance in this observational study, especially as HIV and HSV2 are sexually transmitted infections and so share similar risk factors. We collected data on a range of risk factors, including several measures of sexual risk behavior, and adjusted for all these variables in our analysis. Despite this, there was only slight attenuation of the effect of HSV2 on HIV incidence. We cannot exclude the possibility that there was residual confounding, especially given the limitations of self-reported data on sensitive variables, such as the number and characteristics of sexual partners. However, given the remarkable consistency of our results and those from previous studies, the large odds ratios reported (unlikely to be explained away by residual confounding), and the lack of attenuation of effects after adjustment, it seems likely that the associations represent a causal cofactor effect of HSV2 on HIV acquisition risk.

Second, among those participants who were HSV2 negative at baseline, only 52% were retained at the 36-month follow-up survey and retested for HSV2. This may have resulted in selection bias, as those successfully followed up may differ from those lost to follow-up. However, the main reason for loss to follow-up was having moved permanently out of the study community, and the associations observed among those retained and successfully followed up are likely to be broadly representative of the more stable (nonmigrant) section of the population.

Third, the prospective follow-up was a strength of this study, as it allowed us to estimate the incidence of HSV2 infection and the association of HIV infection with HSV2 status at baseline and during follow-up. Since testing was carried out at only the annual follow-up visits, however, it was still not possible to determine the exact sequence of events in many of the cases where participants seroconverted to HIV and HSV2. Nevertheless, when we examined the subset of cases where this sequence was clearly established, there was a substantial and significant excess of cases in which HSV2 infection preceded HIV seroconversion, adding to the evidence that incident HSV2 infection is a cofactor for HIV acquisition.

Fourth, the Kalon assay is the preferred serologic test for HSV2 for use in sub-Saharan Africa, with 92% sensitivity and 98% specificity [10, 22]. While there may be some false-positive and false-negative results, this measurement bias is likely to be nondifferential, and so measured associations may have been diluted.

Finally, when analyzing associations with behavioral measures and other risk factors, we acknowledge the limitations of self-reported data. We cannot conclude confidently that this reporting bias would be nondifferential, and so the direction of any effect on measured associations is uncertain. Our community-level findings are subject to the usual limitations of ecological analyses.

In conclusion, our study provides strong evidence that the incidence of HSV2 infection in urban communities in Southern Africa is high and that HSV2 infection is an important cofactor for HIV transmission. Differences in the prevalence and incidence of HSV2 help to explain the substantial variation among communities in HIV incidence. Taken together, this study adds to the strong evidence demonstrating a potential role of interventions against HSV2 in reducing HIV incidence in populations with high rates of both these infections. While previous randomized trials showed no effect of acyclovir on HIV infection [23, 24], more recent research on the intimate association between these viruses at cellular level suggests that the null results of those trials likely resulted from therapeutic regimens that were insufficiently potent to significantly disrupt the powerful cofactor effect of HSV2 infection [25, 26]. This highlights the need for effective prophylactic or therapeutic vaccines against HSV2 to help reduce HIV transmission and to decrease the morbidity associated with HSV2. Work continues to develop and test such products [27, 28].

## Supplementary Material

ofae721_Supplementary_Data

## Supplementary Data

Supplementary materials are available at *Open Forum Infectious Diseases* online. Consisting of data provided by the authors to benefit the reader, the posted materials are not copyedited and are the sole responsibility of the authors, so questions or comments should be addressed to the corresponding author.

## Notes

***Acknowledgments*.** We thank all members of the HPTN 071 (PopART) Study Team and all study participants and their communities for their contributions to this research.

***Data availability*.** Requests for the data may be sent to HPTN-Data-Access@scharp.org.

***Financial support*.** This work was supported by the National Institute of Allergy and Infectious Diseases; the US President's Emergency Plan for AIDS Relief; the International Initiative for Impact Evaluation; the Bill and Melinda Gates Foundation; the National Institute on Drug Abuse; and the National Institute of Mental Health.
