# Supplementary material for: Strong Association Between HIV Incidence and Herpes Simplex Virus Type 2 in Zambia and South Africa: Prospective Data From the HPTN 071 (PopART) Trial
Source: Open Forum Infect Dis. 2024 Dec 14;12(1):ofae721. doi: 10.1093/ofid/ofae721 (PMC11733628; doi:10.1093/ofid/ofae721)
Supplement: ofae721_Supplementary_Data [file ofae721_supplementary_data.docx]

Supplementary figure 1: Study flow diagram

38 691 adults aged 18–44 years enrolled at baseline

37 272 (96%) tested for HSV2 seropositivity

20 289 (54%) HSV2 seronegative at baseline

10 539 (52%) retested at the 3 year survey)

1 excluded because date of birth not recorded

10 538 individuals included in the analysis, providing 32 338 years follow up time

Supplementary table 1: Linear association between cluster level HSV2 incidence and cluster level variables

| Variable | Regression coefficient (i.e. increase of HSV2 incidence for a 1 unit increase in the variable) | R^2^ | Adjusted regression coefficient (adjusted for all variables in the table) |
| --- | --- | --- | --- |
| Baseline HSV2 prevalence | 0.21 (0.14, 0.27), p<0.001 | 0.72 | 0.28 (0.08, 0.49) p = 0.011 |
| Baseline HIV prevalence | 0.23 (0.08, 0.37) p = 0.003 | 0.36 | -0.17 (-0.41, 8.1) p = 0.174 |
| Mean number of lifetime sexual partners | 0.03 (0.01, 0.05) p = 0.003 | 0.20 | -0.36 (-2.11, 1.44) p = 0.672 |
| Percentage of males with medical circumcision | -0.14 (-0.20, -0.08) p < 0.001 | 0.55 | -0.03 (-0.10, 0.05) p = 0.461 |

Supplementary table 2: Timing of HSV2 and HIV seroconversion for those who seroconverted to both and for whom the order of seroconversion could be determined

|  | HIV seroconversion | | | |
| --- | --- | --- | --- | --- |
| HSV2 seroconversion |  | Year 1 | Year 2 | Year 3 |
|  | Year 1 | - | 1 | 7 |
|  | Year 2 | 6 | - | 9 |
|  | Year 3 | 0 | 1 | - |

*McNemar’s test: OR = 2.43, 95%CI 1.01-5.86, p=0.047, based on the 24 participants for whom the order of seroconversion could be deduced.
